# Skeletal Site-Specific Response of Jawbones and Long Bones to Surgical Interventions in Rats Treated with Zoledronic Acid

**DOI:** 10.1155/2019/5138175

**Published:** 2019-12-18

**Authors:** Jing Yi Wang, Lei Huo, Ru Qing Yu, Nian Jing Rao, Weijia William Lu, Li Wu Zheng

**Affiliations:** ^1^Discipline of Oral and Maxillofacial Surgery, Faculty of Dentistry, The University of Hong Kong, Prince Philip Dental Hospital, Hong Kong; ^2^Departiment of Orthodontics, The First Affiliated Hospital of Zhengzhou University, Zhengzhou, Henan Province, China; ^3^Department of Orthopedics and Traumatology, Li Ka Shing Faculty of Medicine, The University of Hong Kong, Hong Kong; ^4^Qiongtai Normal University, Hainan, China

## Abstract

Bisphosphonates (BPs) have been extensively used for management of bone diseases with pathologically high resorption. Despite the great clinical benefits, a severe complication known as medication-related osteonecrosis of the jaw (MRONJ) has been reported. It is found that most of the reported MRONJ cases were limited in the jawbones/craniofacial bones instead of long bones. The present study aims to investigate the differential bone response to surgical procedures between jawbones and long bones exposed to BPs. Forty-eight skeletal mature Sprague Dawley female rats were administered oncologic dose of zoledronic acid (ZA) or normal saline for 4 weeks and then subjected to tooth extraction on the mandible and maxilla, and a bone defect creation on the femur. After surgical procedures, ZA or saline treatment were continued until sacrifice at week 2, week 4, and week 8, post-operatively. The samples were subjected to micro-computerized tomography (micro-CT) and histological assessment. Osteonecrosis was only found in jawbones in ZA-treated rats. ZA-treated rats showed significantly higher bone mineral density with greater bone volume in all surgical sites than that in the controls. The length of exposure of ZA did not seem to affect trabecular microstructure, and it only showed higher bone volume and BMD with longer healing time which is expected in the healing process.

## 1. Introduction

Firstly being reported in 2003 (Marx, 2003), medication-related osteonecrosis of the jaws (MRONJ) is one of the potential adverse effects after BPs treatment and has received greater attention more than any other side effects of BPs. One of the most significant characters of MRONJ is the site-specific effect, which is the osteonecrosis tends to occur specifically in maxillofacial bones, the mechanism of which is still unclarified. MRONJ affects the mandible and maxilla with preference for the former, as in mandibles osteonecrosis were found twofold more than that in maxillae [1]. Around half of the MRONJ cases were associated with surgical intervention in the oral cavity [2] or dental diseases [3]. To date, no specific etiological mechanism has been proved to be associated with the pathogenic process in MRONJ. Various assumption and hypotheses have been proposed regarding the etiology of MRONJ, including inhibition of osteoclast activity and over-suppression of bone turnover, suppression of angiogenesis, oral infection, and cell toxicity [4, 5]. However, these hypotheses could not thoroughly explain the exclusive site of occurrence of ONJ. Some studies showed that the high bone turnover rate of jaw bones, together with the increased bone remodeling due to dental surgical operations, resulted in the development of osteonecrosis in this specific site [6]. However, others found that turnover rate of the jawbones was not evidently changed in patients with zoledronic acid or denosumab treatment [7–9].

A minimal number of cases reported atypical fractures of the femur which are related to long-term treatment of bisphosphonates or denosumab [10, 11]. These studies indicated a fracture distinct from the common osteoporosis induced subtrochanteric or femoral shaft fracture, while researchers deduced that the possible mechanisms might be microdamage accumulation, increased mineralization, reduced mineralization heterogeneity, variations in bone turnover rates and reduced vascularity and anti-angiogenic effect related to BPs treatment [12, 13]. Chen et al. [14] demonstrated in a clinical study that long-term use of BPs does not generate a generalized increase in subtrochanteric femoral cortical thickening—which often observed radiographically in patients on long-term bisphosphonates with atypical femur fractures. Except for the above reports, studies on atypical femur fracture and its possible differences or similarity with MRONJ are still lacking. Till now, the skeletal site-specific reactions to BPs treatment associated with trauma have not been fully understood yet. Former studies regarding the comparison of different skeletal site's reaction under surgical intervention toward BPs treatment were in the minority. Ristow et al. [7] investigated the bone turnover rate of femur and jawbones in 90 female cancer patients using scintigraphy with or without BPs treatment. They found that the bone turnover rate was not significantly suppressed by BPs in femur and jawbones. Mandible showed similar bone turnover rate as femur, while the rate in maxilla was significantly higher. Given the fact that clinically peripheral bone seldom went through surgical intervention than that in oral cavity, we aim to investigate the differential bone response to surgical procedures between jawbones and long bones exposed to BPs.

## 2. Materials and Methods

### 2.1. Animal Care and Grouping

A total of forty-eight Sprague Dawley (SD) rats were obtained from the Laboratory Animal Unit (LAU) of Li Ka Shing Faculty of Medicine, the University of Hong Kong. The SD rats were 12-week-old females and weighed from 270 g to 300 g. The animals were kept in a dedicated animal holding facility under the supervision of veterinary. All animals were housed in an indoor environment at a temperature of 20°C ± 5°C in a 12 : 12-h light–dark circle with free access to water and standard rodent diet (Irradiated, PMI, USA). The animal study was approved by the Committee on Use Live Animal for Teaching and Research, the University of Hong Kong (CULATR 3775-15).

Forty-eight rats were randomly assigned into two groups with twenty-four rats in each. Animals in Group ZA received intraperitoneal injection (*i.p*.) of zoledronic acid (Zometa, Novartis, Switzerland. 0.066 mg/kg) dissolved in 0.2 ml sterile saline three times per week. This dosage scheme corresponds to 4 mg/60 kg drug dosages monthly for cancer patients with skeletal complications [15]. However, Group C (control group) received an equivalent amount of normal saline *i.p.* three times per week. The administration of ZA or saline continued from baseline until the sacrifice of the animals.

**Figure 1:**
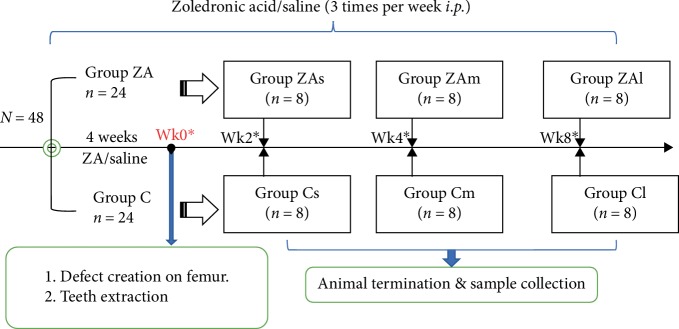
Flow chart of grouping and treatment of animals. A total of forty-eight rats are randomly assigned into ZA-treated group (Group ZA) and control group (Group C). Each group is further divided into three subgroups based on the length of treatment. Zoledronic acid (0.066 mg/kg) is administered intraperitoneally three times per week from the start of the experiment until sacrifice. At the end of the fourth week, all animals receive surgical intervention including a defect creation on the right femur and lower first molar and upper first molar extraction. Animals are sacrificed at two weeks, four weeks and eight weeks after surgery. ^∗^The timepoints were counted starting from the time of the surgery. Before surgical intervention, four weeks of ZA/normal saline is administered.

After four weeks (which roughly equivalent to 2.5 human years of administration [16]) of ZA/saline administration, all animals received surgical intervention. These two groups of animals were further divided into six subgroups which were subjected to different length of exposure (LoE) with the treatment of ZA/saline sustained until sacrifice of the animals. The rats in Group ZAs (short-term experiment group, *n*=8) and Group Cs (short-term control group, *n*=8) were sacrificed at the second week after surgery. Likewise, animals of Group ZAm (medium-term experiment group, *n*=8) and Group Cm (medium-term control group, *n*=8) were sacrificed at the fourth week and Group ZAl (long-term experiment group, *n*=8) and Group Cl (long-term control group, *n*=8) were sacrificed at the eighth week after surgery (Figure 1).

### 2.2. Surgery

All the animals received right femur defect creation and right lower and upper first molar extraction. Using a Ø2.0 trephine bur (Trephine Drill 2 mm (3 mm OD)  × 10 mm Barrel, ForeverGreen, Hong Kong) in a low-speed motor handpiece, a round defect with a diameter of 3.0 mm in the lateral aspect of the right femur was created through the cortical bone. The whole thickness of the cortical bone was removed until marrow cavity was reached without further destroying the opposite side of the cortical bone.

After the operation on the right femur, the right maxillary and mandibular first molars were extracted using a standard protocol. A dental explorer was used as a gingival separator to disconnect the surrounding gingiva of the molar. The right mandibular and maxillary first molars were removed using a children's extracting forceps. The extraction sites were left open, and a small cotton wool roll was pressed onto the extraction socket until bleeding stops.

#### 2.2.1. Post-Operative Care

Post-operatively, the rats were given Enrofloxacin (Baytril 5%-enrofloxacin 250 ml, Bayer, Leverkusen, Germany) adding in drinking water, with 2 ml in 500 ml water in q72 hours. Mobic (Meloxicam, 0.6 mg/kg) and Temgesic (Buprenorphine, Reckitt Benckiser Health, Slough, UK, 0.05 mg/kg) were administered subcutaneously (s/c) for pain relief. Animals were closely monitored until alert and drinking. Gel diets were given q72 hours post-operatively. The animal's clinical condition, weight, and food consumption were carefully monitored. Sutures were removed at the seventh day after the operation.

#### 2.2.2. Micro-Computerized Tomography

All collected specimens were scanned with a micro-computerized tomography (micro-CT) system (SkyScan1076; Bruker, Kontich, Belgium) according to the manufacturer's instructions in Department of Orthopaedics and Traumatology, Li Ka Shing Faculty of Medicine, the University of Hong Kong. The samples were scanned at an energy of at 88 kV and 100 *µ*A intensity with a resolution of 8.665 *µ*m pixel with a filter of 1.0 mm-thickness aluminum. Reconstruction was done using the SkyScan NRecon program (Version, 1.7.3.0, SkyScan, Kontich, Belgium). The data sets obtained after reconstruction were loaded into the SkyScan CT-analyzer software (CTAn, version 1.12.0, SkyScan, Kontich, Belgium) for further assessment and three-dimensional images obtained by the CTvox software (Version 3.3, SkyScan, Kontich, Belgium).

#### 2.2.3. Region of Interest

**Figure 2:**
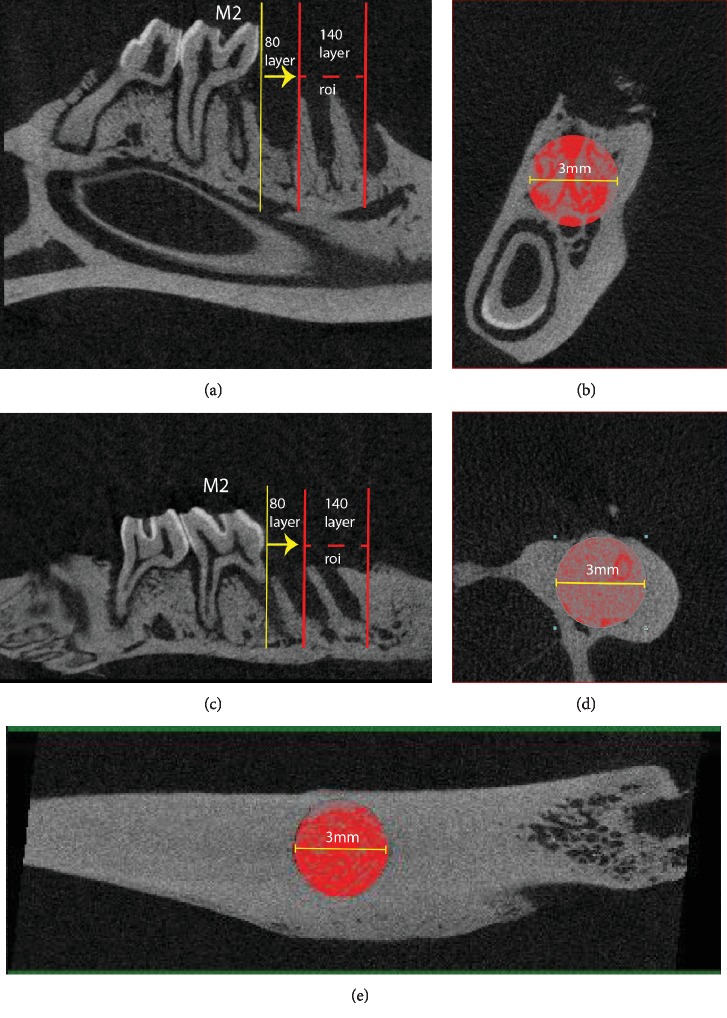
Selection of volume of interest in mandible extraction socket (a) sagittal plane, (b) coronal plane, maxilla extraction socket (c) sagittal plane, (d) coronal plane, and femur defect site (e) sagittal plane.

The whole specimens were carefully observed for any bone sequestra or periosteal reaction before selection of Volume of interest (VOI). A cylindrical region in the trabecular bone of the first molar (M1) socket was selected to be the region of interest in maxilla and mandible. Sagittally, we chose 80 layers at the coronal plane starting from the mesial surface of the second molar crown (M2) to the alveolar socket of M1 and set for the bottom layer of the VOI and then continued selection for 140 layers and set for the top layer of the VOI. Coronally, a round region of interest (ROI) of 3.00 mm in diameter was set among the trabecular bone in these 140 layers (Figure 2). For femur defect, ROI was defined as 140 layers from the level of cortical bone with a 3.0 mm diameter circle covering the defect size (Figure 2).

The trabecular microstructure was assessed using the parameters as described in Table 1.

#### 2.2.4. Histopathology Assessments

After completion of micro-CT scanning, the specimens were trimmed to appropriate size and rinsed in tap water for two hours. After rinsing, samples were decalcified in 12.5% ethylenediaminetetraacetic acid (EDTA) (pH = 7.2) at room temperature for three months. Once decalcified, all specimens were embedded and sliced in a thickness of 5 *µ*m using semiautomated microtome (Leica Biosystems, Wetzlar, Germany). Goldner trichrome staining was performed using a standard protocol [17].

Microscope sections were viewed under Eclipse LV 100POL (Nikon Corporation, Japan), and images were taken using DS-Ri1 high-resolution microscope camera (Nikon Corporation, Japan). All measurements were standardized and carried out by two well-trained examiners in Centralized Research Laboratory, Faculty of Dentistry, the University of Hong Kong. Osteonecrosis was determined as contiguous empty osteocytic lacunae (continued empty lacunae up to 5 in a row) in trabecular bone together with the loss of osteocytes. Semiquantitative analysis was carried out using ImageJ software (version 1.51 s, National Institutes of Health, Bethesda, USA). Three high power fields were randomly selected, and empty/viable lacunae were calculated.

#### 2.2.5. Statistical Analysis

The data were presented as mean + SD and were analyzed using IBM SPSS statistic software (version 24.0, IBM Crop, Armonk: NY, USA). Two-way ANOVA test was conducted to examine the effect of zoledronic acid on bone mineral density (BMD) in surgically treated jawbones and long bones in different time points. Where applicable, further assessment of Bonferroni's multiple comparison post hoc or independent-samples *t*-test were used. Kruskal–Wallis test and Mann–Whitney test were used for non parametric data. The significance level was set at *p* < 0.05.

## 3. Results

### 3.1. Clinical Observation

All the rats completed the experiment uneventfully. Among all groups, six rats were observed with femur fracture, with two in Group Cm, one in Group Cs, one in Group ZAm, and two in Group ZAs. Three cases were observed with bone exposure (3/24; one in maxilla, two in mandible) in ZA-treated rats. Only one case in control group presented with exposed bone (1/24; in maxilla). Soft tissue fenestrations were observed in four cases (4/24) in the maxilla, and four (4/24) in the mandible in ZA-treated group, while two cases (2/24) in the mandible in control group. There were no soft tissue fenestrations or bone exposure observed in femur region in both ZA-treated and control groups.

### 3.2. Micro-CT Examination

**Figure 3:**
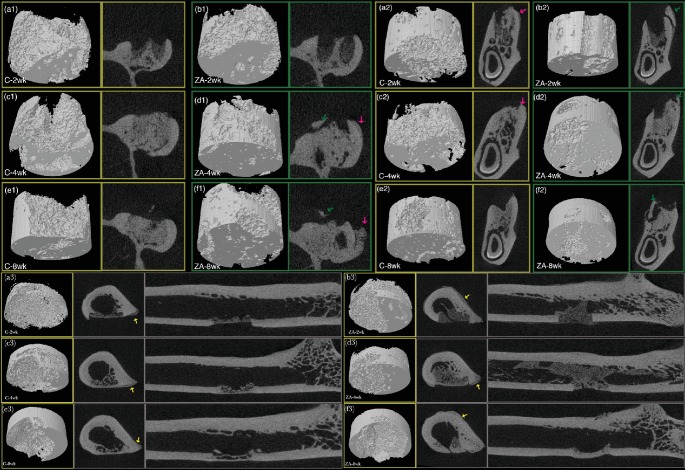
Micro-CT 3D and 2D cross-sections of maxilla (a1-f1), mandible (a2-f2), and femur defect areas (a3-f3). 3D VOI, transverse and coronal plane cross-sectional images of control groups and ZA-treated group at week 2, week 4, and week 8. Red arrow points to periosteal reaction. Green and yellow arrows indicate bone sequestrum. 3D cylindrical sub-volume covered the defect area is the VOI for assessment of BMD and trabecular indices.

Micro-CT analysis was used to better identify bone sequestrum and provide a three-dimension analysis for changes of bone density and microarchitecture (Figure 3). Periosteal reaction and bone sequestrum formation were observed.

#### 3.2.1. Bone Mineral Density

The average bone mineral density (BMD) of mandibular and maxillary extraction sockets was higher than that in femur defect area compared between inter-groups and intra-groups (Table 2).

In femur defect healing site, a steady increase in bone mineral density was noted in ZA-treated group and control group. Inter-group comparison of different time points showed higher BMD with longer length of exposure (LoE) (*F*(2, 36)=47.774, *p* < 0.001). However, no significant difference was found between ZA-treatment group and control group in BMD. The interaction effect of ZA treatment and LoE was not significant as well. Within treatment groups and control groups, significant difference of BMD was found as longer LoE with higher BMD (*p* < 0.001); however intra-group comparison of ZA and control groups, only a marginal significance was found between long-term ZA-treated group and long-term control group (*p*=0.049).

In mandible tooth extraction site, an increase in bone mineral density after surgery in control group was also found. However, in ZA-treated group, the bone mineral density slightly decreased at 4 weeks after surgery compared with 2 weeks after surgery and increased to the highest in the 8 weeks group. Both two independent variables (ZA treatment and length of exposure) showed significant difference, yet the interaction effect was not significant. The main effect for treatment yielded an *F* ratio of *F*(1, 42)=7.858, *p* < 0.01, indicating a significant difference between ZA treatment (*M*=0.856, *SD*=0.09) and control group (*M*=0.798, *SD*=0.07). The effect of LoE was also found significant, which yielded an *F* ratio of *F*(2, 42)=10.251, *p* < 0.001. In inter-group comparison between ZA and control groups, significant difference of BMD was found in long-term groups between ZA treatment and control (*p* < 0.01), with ZA-treated group exhibiting higher bone mineral density. Intra-group comparison showed significant difference comparing long-term ZA group with medium-term and short-term (*p* < 0.01), while in control groups, significance was found only between long-term group and short-term group (*p* < 0.01).

In maxilla tooth extraction site, the BMD value experienced a steady increase with longer LoE in both ZA-treated group and control group. Only LoE factor showed significant difference with an *F* ratio of *F*(2, 42)=27.919, *p* < 0.001. No significant difference was found in regard to treatment and the interaction between time duration and treatment. Pairwise comparisons in three time points showed no significant difference between ZA-treated group and control group. However, within treatment factor, significant increase of BMD was found with longer treatment duration when comparing the short-term group with long-term group (*p* < 0.05).

#### 3.2.2. Trabecular Microarchitecture

The main effect of ZA treatment and treatment duration, and their interaction in bone microstructure are listed in Table 3.

Intra-group comparison among different time points had no significant differences in femur, mandible, and maxilla. However, the inter-group comparison between ZA treatment and control revealed that in mandible, Tb.N was significantly higher in medium-term and long-term treatment group, and Tb.Sp were significantly lower in medium-term and long-term treatment group. The fraction of bone volume out of the total volume (BV/TV) in medium-term and long-term ZA-treated mandibular extraction socket both showed significantly higher compared with the corresponding control group. This is correlated with a reduction in the bone marrow space in the mandibular bone and between the roots of the first molar.

Overall, in femur defect, the trabecular thickness and trabecular number per mm^2^ increased with the increasing bone volume fraction, while trabecular separation decreased. This indicated the healing of femur defect manifested as both increasing of trabecular number and thickness. Yet in mandible extraction sites, Tb.Th, Tb.N, and Tb.Sp reached plateau (though slightly increased for Tb.Th and Tb.N and slightly decreased for Tb.Sp in eight weeks group) in week 4 for control group, while for ZA-treated group, the remodeling continued to the eighth week after surgery. In maxilla extraction sites, changes of trabecular thickness through time was not significant in ZA-treated group. With increasing of bone volume in the healing process, the trabecular number increased steadily with the decreasing of trabecular separation. This indicates that the bone healing of maxilla under ZA treatment mainly resulted from the increased number of trabecular bone rather than widening of trabecular thickness, which in control group, the increase of bone mass was from both sides.

### 3.3. Histopathology Examination

**Figure 4:**
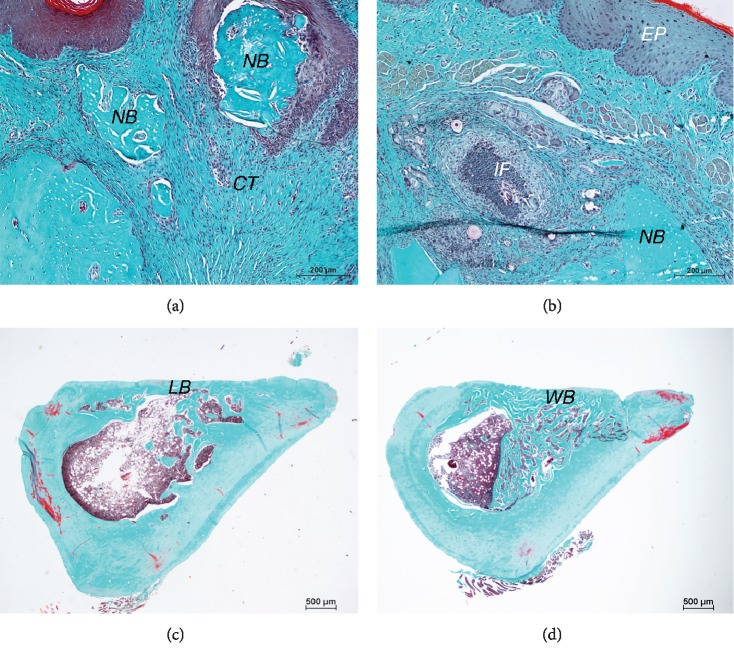
Representative histological findings of osteonecrosis, inflammation and femur defect healing. (a) Bone sequestra found in ZA-treated mandible. (b) Soft tissue inflammation found in ZA-treated mandible. Scale bar = 200 *µ*m. (c) Femur of long-term control group, formation of cortical bridge at defect site. (d) Femur of long-term ZA group, spongy bone without maturation into cortical bone in defect site. Scale bar = 500 *µ*m. NB: necrotic bone; CT: connect tissue; IF: inflammatory infiltration; EP: epithelium; LB: lamellar bone; WB: woven bone.

In femur specimens, histological osteonecrosis (which is defined as continued empty lacunae up to 5 in a row) [18] was only observed in one sample in medium-term ZA-treated group and one in medium-term control group. In short-term groups, ZA-treated femur showed more connected bone structures than that in control group. Four weeks after surgery, the medium-term groups both displayed an increased volume of connected bone structures at the defect area, while ZA-treated femur showed more woven bone extending into the medullary cavity (Figure 4). At the eighth week post-operation, a nearly developed cortical bone bridge has been observed on femur defect in most of the control group, while ZA-treated femur showed less developed cortical bone and more woven bone extending into the medullary cavity.

In mandibular specimens, two weeks after surgery, the extraction socket of ZA-treated sample, as well as control sample was filled with fibrous tissue with some newly formed viable woven bone. Eight weeks after extraction, the woven bone was almost entirely replaced by lamellar bone or trabecula in the socket in most of the control samples, which showed as normal wound healing and bone remodeling. ZA-treated mandible showed delayed bone remodeling and bone necrosis in some of the cases. Histological osteonecrosis precedes clinical manifestation; there were only 1/6 of histological osteonecrosis found with clinical bone exposure in the maxilla and 2/14 in the mandible. Histological osteonecrosis was observed mostly at the top of the lingual or buccal side of the cortical bone in ZA-treated groups at all three time points, while few short-term control samples showed clustered empty lacunae in cortical area adjacent to extraction socket as well. Randomly diffused non viable osteocytes were also found in mandible samples of control groups.

In maxilla specimens, histological osteonecrosis was also found at the top of lingual or buccal maxillary cortical bone in ZA-treated groups at different time points, while only one long-term control sample was found with osteonecrosis.

The results of histological assessment of fraction of empty lacunae out of viable lacunae are shown in Table 4.

## 4. Discussion

The prolonged duration of drug administration has been well documented in relation to the development of MRONJ. The drug accumulating effect on different sites of skeletal bones might play a part in the site-specific effect of MRONJ. Studies showed that BPs exerted direct toxic effects on oral epithelial cells and fibroblasts when accumulated at sufficient concentrations in bone tissue [19–22]. This toxic effect further led to delayed soft tissue healing and secondary infection and resulted in necrosis of the underlying bone. However, a number of MRONJ cases occurred prior to soft tissue infection and injury [23]. Thus, the causal relationship between infection and MRONJ is still a matter of debate [24–26].

Tooth extraction is believed to be the most common predisposing event of MRONJ. A previous systematic review reported that around 61.7% of patient with MRONJ had a history of tooth extraction [27], while among cancer patient receiving intravenous BPs treatment, the estimates of osteonecrosis after tooth extraction range from 1.6% to 14.8%. [27–30]. The pathogenesis of MRONJ are multifactorial yet not fully explained; meanwhile, the bone-specific character of MRONJ has not been well investigated.

Given the fact a large portion of MRONJ cases had a history of tooth extraction/invasive intervention in the oral cavity, it is the oral cavity that takes up the most invasive operation rather than other parts of skeletal sites. We designed an animal model with surgical intervention at jawbones as well as long bones simultaneously. To our knowledge, animal models for MRONJ following trauma/tooth extraction have been established in many studies [31–35], yet a comparison of surgical intervention in jawbones and peripheral bone has seldom been reported. In the present study, a femur defect of 3 mm in diameter was created along with tooth extraction to provide the same surgical intervention to mandible, maxilla, and femur. ZA treatment was continued for 2, 4, and 8 weeks after the surgery. In addition to the clinical and macroscopic examination, micro‐CT examination was used to assess bone sequestration and periosteal reaction as it provides 3D and 2D images in three different planes. Although the MRONJ staging system is based on clinical manifestations without radiological evaluation [36, 37], there are still characteristics that clinical examination could not access. Previous investigations have reported that CT examination of patients with MRONJ showed periosteal reaction, cortical perforation, periosteal bone deposition, mandibular fractures, *etc.,* [38]. A recent study using CT imaging on 74 Japanese patients who suffered from ARONJ/MRONJ showed that most lesions of ONJ were both lytic and sclerotic. Meanwhile, observative results of the same study revealed that the size of the sequestra were mostly small with some of them exhibited periosteal reaction, while cortical bone perforation was more commonly seen in buccolingual or buccal cortical bone other than lingual cortical bone [39]. Cone beam computed tomography (CBCT) analysis has also shown the lesions in MRONJ with thicker cortical bone and more sclerotic bone marrow [40, 41].

In the present study, micro-CT examination demonstrated a number of cases in ZA-treated/control groups exhibiting periosteal reaction. Bone sequestra were also observed in five cases in maxilla and eight cases in mandible in ZA-treated groups. There was only one case of sequestrum formation found in control group in the mandible. No necrotic bone was observed in the micro-CT examination in femur region. The size of the sequestra in mandibles and maxillae were also found to be small and irregular, mostly on the buccal or lingual side of the cortical bone.

Osteonecrosis is mostly found in jawbones, yet atypical femur fracture has been reported in many BPs-treated patients as well. This raises our attention that femur and jawbones react differently towards BPs treatment. Some clinical studies suggested that long-term BPs exposure leads to the formation of small transverse crack in the femur that stimulates periosteal reaction which further leads to breaking or flaring in an attempt to heal the fracture [10, 14].

In the present study, there is no significant association between the treatment duration and ZA treatment in inducing osteonecrosis in femur, mandible, and maxilla. BMD value had significantly increased with longer healing time in femur defect, while no significant difference between treatment and control in femur at every time point except for a marginal significance higher of BMD in long-term ZA compared with long-term control (*p*=0.049). Similar results of BMD were found in mandible as well. ZA treatment before and after surgical intervention resulted in increased trabecular bone volume in medium-term and long-term treated femur and mandible, while no significant difference was found in maxilla. Between different skeletal sites, femur defect region showed lower BMD than that of jawbones. This might be due to the different surgical intervention method, which resulted in bone remodeling and healing for direct bone defect in femur, while in jawbones, remodeling and healing for tooth extraction sockets. Among all the parameters, femur defect exhibited the most substantial changes in BMD and bone volume, which indicates a steady healing process. As for bone microstructure, BV/TV, Tb.N, Tb.Th, and Tb.Sp were analyzed in the present study as they are acknowledged as the minimal set of variables for describing trabecular bone morphometry [42]. ZA treatment resulted in significant increased trabecular number and decreased trabecular separation in medium-term and long-term treated mandible; similar results were found in maxilla and femur but not significant. These micro-CT results showed that normal bone remodeling was delayed in ZA-treated group in both femur and jawbones after surgical intervention. However, similar to clinical findings, bone sequestrum has only been found in jawbones.

Histological results corresponded with micro-CT examination. We found more formation of woven bone in femur defect, yet slower maturation into cortical bone in femur defect of ZA-treated group compared with control group. Interestingly, a research group recently studied the properties of trabecular bone tissue in the femoral head in patients who experienced a femoral neck fracture [43, 44]. These patients in BPs-treated groups had taken weekly oral alendronate for 1–9 years with an average of 3 years. It was assessed using synchrotron imaging and compression mechanical testing that femur samples of BPs-treated patients showed significantly lower strength and modulus compared with control. Meanwhile, BPs-treated patients also exhibited higher microcrack density and volume compared to control.

Clustered empty lacunae were found mostly at the top of lingual or buccal wall of the cortical bone, which is the most common location found in MRONJ cases. No noticeable signs of osteonecrosis were found in all femur defect. However, the ratio of empty lacunae was significantly higher in ZA-treated mandible and maxilla. This result agrees with the site-specific character of MRONJ. Interestingly in ZA-treated mandible and maxilla, regardless of higher occurrence of osteonecrosis and higher number of empty lacunae, most samples were still found with regular volume of new bone tissue extending from the bottom of the extraction socket to the top, on the contrary, only a few samples were found with obviously delayed healing on the site of the empty socket. However, osteonecrosis happened on these delayed healing animals as well as others, even in some short-term control animals. This can be a short-term histological post-operative reaction.

**Figure 5:**
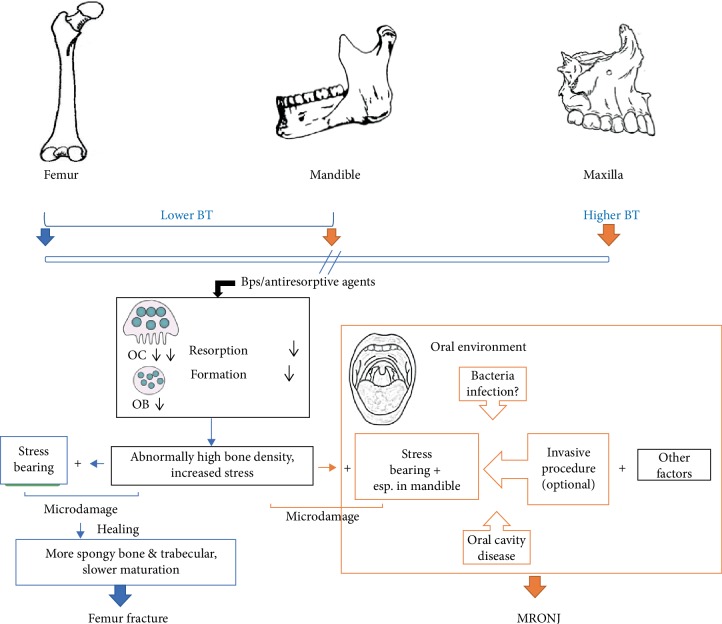
Schematic illustration of known and putative differences of BPs effect on long bones and jawbones. BT: bone turnover; OC: osteoclast; OB: osteoblast. Femur and mandible show relatively lower bone turnover rate than maxilla. The activity of osteoclasts is inhibited which lead to inhibition of osteoblasts activity under BPs treatment. This further results in abnormally high bone density and increased stress in the bone tissue. During healing process of microdamage, more spongy bone and woven bone are formed and slowly mature into lamellae, which lead to femur fracture. Same as with femur situation, jawbones also bear the increased stress in bone tissue, and mandible is likewise a stress-bearing anatomy. However, different from femur, jawbones succumb to ONJ under BPs treatment. Factors such as infection and oral diseases may play a more crucial role in the site-specific character of MRONJ.

As reported by Ristow et al. [7], femur and mandible showed relatively lower bone turnover rate than maxilla. Along with the findings of the present study, the schematic illustration of known and putative differences in effects of BPs on peripheral bone and jawbones is summarized in Figure 5. Under the administration of BPs or other antiresorptive agents, the activity of osteoclasts is inhibited which lead to inhibition of osteoblasts activity. This further results in abnormally high bone density and increased stress in the bone tissue. Within the stress-bearing area in femur, together with increased stress, microdamage occurs. During healing process of microdamage, more spongy bone and woven bone are formed and slowly mature into lamellar bone, which lead to femur fracture. Same as with femur situation, jawbones also bear the increased stress in bone tissue, and mandible is likewise a stress-bearing anatomy which would invariably withstand load and microdamage [45]. However, different from femur, jawbones succumb to ONJ under BPs treatment. Kim et al. [46] investigated the role of microcrack accumulation in BRONJ in ovariectomized rats which underwent tooth extraction. They found that both number of cracks and number of crack length in the BPs group were greater than those in the control group, of which they concluded that the accumulation of unrepaired microcracks was significantly related to the onset of MRONJ. The difference between peripheral bone and jawbones, besides their modes of ossification, is the open environment of the oral cavity. The findings of our study showed that invasive procedure is not the determinative factor for osteonecrosis, adding with reports in previous pieces of the literature that bone turnover rate between femur and mandible is not significantly different. Thus, factors such as infection, oral diseases, angiogenesis inhibition may play a more crucial role in the site-specific character of MRONJ.

## 5. Conclusions

The present study showed that zoledronic acid treatment did have site-specific effect on surgically treated jawbones verses long bones, where necrosis only occurred in the jaw. Osteonecrosis occurred more frequently at the site of the mandibles than maxillae. However, there is no noticeable difference concerning the changes in bone tissue healing under ZA treatment between femur and jawbones. The length of exposure of ZA did not seem to affect trabecular microstructure and fraction of empty lacunae/viable lacunae; it only showed higher bone volume and BMD with longer healing time which is expected in the healing process.

This study has the limitation that the femur and jawbones did not go through precisely the same surgical procedure, which might render differences in the bone healing process. However, to better mimic the real situation, we chose tooth extraction procedure rather than creating a same sized defect in jawbones. The latter case will be more challenging to produce and not in accordance with clinical reality.

## Figures and Tables

**Table 1:** Output parameters for micro-CT examination of trabecular bone.

Parameter	Abbreviation (unit)	Meaningfulness
Bone volume/tissue volume	BV/TV (%)	The percentage of trabecular bone volume in the selected volume of interest
Bone surface/bone volume (specific bone surface)	BS/BV (mm^−1^)	The ratio of solid surface to bone volume which characterizing the thickness and complexity of trabecular structures
Bone surface/tissue volume	BS/TV (mm^−1^)	The ratio of solid surface in the selected volume of interest
Trabecular thickness	Tb.Th (mm)	The thickness of trabecular structures
Trabecular number	Tb.N (mm^−1^)	The number of traversals across a trabecular structure per unit in selected VOI
Trabecular separation	Tb.Sp (mm)	The thickness of space in selected VOI, in which higher value indicates reduced connectivity
Bone mineral density	BMD (g/cm^3^)	The mass of bone mineral per volume of bone

**Table 2:** Effect of zoledronic acid on bone mineral density (mean ± SD) in femur defect, and mandibular/maxillary extraction socket.

BMD *(g/cm^3^)^∗^*	2 wk	4 wk	8 wk
*Femur*			
ZA	0.32 ± 0.09	0.57 ± 0.18	0.79 ± 0.06^∗^
Control	0.35 ± 0.16	0.54 ± 0.06	0.68 ± 0.06
*Mandible*			
ZA	0.84 ± 0.05	0.83 ± 0.09	0.93 ± 0.05^∗∗^
Control	0.72 ± 0.07	0.81 ± 0.07	0.85 ± 0.03
*Maxilla*			
ZA	0.75 ± 0.05	0.84 ± 0.03	0.91 ± 0.08
Control	0.67 ± 0.09	0.84 ± 0.13	0.93 ± 0.07

^∗^Differences were considered significant at *p* < .05, ^∗^*p* < 0.05, ^∗∗^*p* < 0.01.

**Table 3:** Micro-CT assessment of morphology of jawbones and femur.

*p*-value^∗^	ZA treatment			LoE^†^			Interactions		
	Mandible	Maxilla	Femur	Mandible	Maxilla	Femur	Mandible	Maxilla	Femur
BV/TV (%)	0.000	0.069	0.000	0.000	0.000	0.000	0.251	0.212	0.054
BS/BV (mm^−1^)	0.000	0.077	0.400	0.000	0.000	0.000	0.703	0.003	0.169
BS/TV (mm^−1^)	0.015	0.451	0.011	0.000	0.008	0.023	0.214	0.209	0.086
Tb.Th (mm)	0.102	0.403	0.000	0.009	0.100	0.000	0.491	0.037	0.000
Tb.N (mm^−1^)	0.040	0.008	0.008	0.823	0.008	0.000	0.030	0.002	0.038
Tb.Sp (mm)	0.003	0.148	0.256	0.538	0.065	0.000	0.032	0.835	0.052

^∗^Significant (*p*-value) of trabecular indices assessed by two-way ANOVA. Differences were considered significant at *p* < .05, † LoE: length of exposure.

**Table 4:** Histological assessment of fraction of empty lacunae out of viable lacunae.

Empty/viable^†^	Short-term	Medium-term	Long-term
*Femur*			
ZA	0.09 ± 0.08	0.08 ± 0.09	0.14 ± 0.07
Control	0.18 ± 0.29	0.04 ± 0.03	0.08 ± 0.06
*Mandible*			
ZA	0.52 ± 0.66^∗^	0.22 ± 0.26^∗^	0.30 ± 0.57^∗^
Control	0.10 ± 0.12	0.04 ± 0.03	0.06 ± 0.06
*Maxilla*			
ZA	0.28 ± 0.38	0.12 ± 0.07^∗∗∗^	0.23 ± 0.26^∗∗∗^
Control	0.10 ± 0.11	0.02 ± 0.02	0.03 ± 0.03

^†^Differences were considered significant at *p* < 0.05, ^∗^*p* < 0.05, ^∗∗∗^*p* < 0.001.

## Data Availability

The datasets used to support the findings of this study are available from the corresponding author upon request.
